# Deep learning to estimate impaired glucose metabolism from Magnetic Resonance Imaging of the liver: An opportunistic population screening approach

**DOI:** 10.1371/journal.pdig.0000429

**Published:** 2024-01-16

**Authors:** Lea J. Michel, Susanne Rospleszcz, Marco Reisert, Alexander Rau, Johanna Nattenmueller, Wolfgang Rathmann, Christopher. L. Schlett, Annette Peters, Fabian Bamberg, Jakob Weiss

**Affiliations:** 1 Department of Diagnostic and Interventional Radiology, University Hospital Freiburg, Freiburg, Germany; 2 Department of Epidemiology, Institute for Medical Information Processing, Biometry, and Epidemiology, Ludwig-Maximilians-University Munich, Munich, Germany; 3 Institute of Epidemiology, Helmholtz Zentrum München, German Research Center for Environmental Health, Neuherberg, Germany; 4 German Center for Cardiovascular Research (DZHK), partner site Munich Heart Alliance, Germany; 5 Medical Physics, Department of Radiology, Medical Center—University of Freiburg, Freiburg, Germany; 6 Institute for Biometrics and Epidemiology, German Diabetes Center, Leibniz Center for Diabetes Research, Heinrich Heine University Düsseldorf, Düsseldorf, Germany; German Center for Diabetes Research, München-Neuherberg, Germany; 7 German Center for Diabetes Research (DZD), partner site Neuherberg, Neuherberg, Germany; University of Washington, UNITED STATES

## Abstract

**Aim:**

Diabetes is a global health challenge, and many individuals are undiagnosed and not aware of their increased risk of morbidity/mortality although dedicated tests are available, which indicates the need for novel population-wide screening approaches. Here, we developed a deep learning pipeline for opportunistic screening of impaired glucose metabolism using routine magnetic resonance imaging (MRI) of the liver and tested its prognostic value in a general population setting.

**Methods:**

In this retrospective study a fully automatic deep learning pipeline was developed to quantify liver shape features on routine MR imaging using data from a prospective population study. Subsequently, the association between liver shape features and impaired glucose metabolism was investigated in individuals with prediabetes, type 2 diabetes and healthy controls without prior cardiovascular diseases. K-medoids clustering (3 clusters) with a dissimilarity matrix based on Euclidean distance and ordinal regression was used to assess the association between liver shape features and glycaemic status.

**Results:**

The deep learning pipeline showed a high performance for liver shape analysis with a mean Dice score of 97.0±0.01. Out of 339 included individuals (mean age 56.3±9.1 years; males 58.1%), 79 (23.3%) and 46 (13.6%) were classified as having prediabetes and type 2 diabetes, respectively. Individuals in the high risk cluster using all liver shape features (n = 14) had a 2.4 fold increased risk of impaired glucose metabolism after adjustment for cardiometabolic risk factors (age, sex, BMI, total cholesterol, alcohol consumption, hypertension, smoking and hepatic steatosis; OR 2.44 [95% CI 1.12–5.38]; p = 0.03). Based on individual shape features, the strongest association was found between liver volume and impaired glucose metabolism after adjustment for the same risk factors (OR 1.97 [1.38–2.85]; p<0.001).

**Conclusions:**

Deep learning can estimate impaired glucose metabolism on routine liver MRI independent of cardiometabolic risk factors and hepatic steatosis.

## Author summary

Type 2 diabetes presents a significant global health challenge, affecting an estimated 536.6 million individuals, comprising 10.5% of the adult population worldwide. Alarmingly, nearly half of those affected remain undiagnosed, unaware of their elevated risk of morbidity and mortality. Although cheap and accurate tests exist, the high number of undiagnosed individuals demonstrates the need for novel approaches. Especially in medical imaging, we have large amounts of data acquired every day, yet most of the information embedded in the scans remains unused. With deep learning, it is now possible to automatically extract this information in an opportunistic fashion. For this study a fully automatic deep learning framework was developed to investigate the association between radiomic shape features of the liver and impaired glucose metabolism in a sample from a population-based cohort of individuals with prediabetes, type 2 diabetes and healthy controls without prior cardiovascular diseases. Our results demonstrate that the proposed deep learning framework allows for identifying individuals at risk for impaired glucose metabolism on routinely acquired MRI of the liver. These findings indicate the potential for opportunistic screening to identify individuals at risk of type 2 diabetes to improve screening and early diagnosis, to reduce long-term complications and economic expenses.

## Introduction

Type 2 diabetes is defined as a relative insulin deficiency resulting from defects in the pancreatic betta cells and insulin resistance in target organs [1]. During the past decades, the prevalence has dramatically increased due to rapid urbanisation and a more sedentary lifestyle [2,3,4]. Currently, the global prevalence of diabetes counts 536.6 million people or 10.5% of the adult population and is considered to further increase with an estimate of 783.2 million diseased individuals by 2045 [4]. This is of great importance, as the management of type 2 diabetes and its complications is placing a substantial economic burden on the healthcare system [5]. For example, Jacobs et al. revealed, that in Germany, 10% of the total salutary health insurance expenses, in total 16.1 billion euros, were attributed to the medical care of type 2 diabetes in 2017 [6]. In the same year, the total expenditure on diabetes treatment in the United States was 327 billion dollars [7,8]. In addition, there is a high number of individuals with undiagnosed diabetes, who are not aware of their increased risk of morbidity and mortality because disease onset is typically subclinical and asymptomatic [9]. Although dedicated tests for screening and early diagnosis are available, such as glycated haemoglobin (HbA1c) or oral glucose tolerance testing, correct interpretation may be confounded with the risk of reduced accuracy [10,11,12,13]. Further, non-invasive risk scores and biomarkers such as the QDScore or DPoRT are valuable tools to identify individuals at high risk of type 2 diabetes in the general population but have been shown to perform sub-optimally at an individual level [14,15,16,17]. This demonstrates the need for novel approaches to improve screening and early diagnosis to reduce long-term complications and economic expenses.

Recent developments in deep learning–a form of artificial intelligence, have opened a new window for high-throughput quantitative analysis. This is particularly true for medical imaging, where large amounts of data are acquired every day, yet most of the information embedded in the scans remains unused due to time and equipment constraints. With deep learning, it is now possible to automatically extract this information in an opportunistic fashion from scans acquired in daily care with the potential to improve clinical decision-making and patient management.

Here, we developed a fully automatic deep learning framework to quantify radiomic shape features of the liver on routine MRI to estimate states of impaired glucose metabolism in a sample from a population-based cohort. We hypothesize, that MRI of the liver, a key organ in glucose metabolism and homeostasis, contains features indicative of impaired glucose metabolism independent of traditional cardiometabolic risk factors and hepatic steatosis and allows for opportunistic screening of individuals at risk of impaired glucose metabolism.

## Material and methods

### Study population

All analyses were performed using data from the Cooperative Health Research in the Region of Augsburg (KORA) MRI study, a cross-sectional MRI study nested in the prospective epidemiological cohort of the KORA main study [18,19]. The study population of the KORA-MRI study was recruited between June 2013 and September 2014 and consisted of 400 participants who underwent a multiparametric whole-body MRI study protocol as previously described in detail [19]. Subjects were eligible if they met the following inclusion criteria: consent to undergo whole-body MRI, no prior cardiovascular disease, and a standardized assessment of glucose metabolism.

All participants gave written informed consent, and the study protocol was approved by the ethics committee of the Bavarian Chamber of Physicians and the institutional review board of the Ludwig-Maximilians-University Munich and complied with Helsinki’s declaration of human research.

### MRI protocol

Whole-body MRIs were acquired on a 3T MRI scanner (Magnetom Skyra, Siemens Healthcare, Erlangen, Germany) in supine position using an 18-channel body surface coil and a table mounted spine matrix coil. The complete MRI protocol has been described previously [19]. For the current study, only the two-point T1-weighted isotropic Volumetric interpolated breath-hold examination (VIBE)—Dixon gradient-echo sequence was used, which was acquired with the following acquisition parameters: slice thickness: 1.7 mm, in-plane resolution 1.7x1.7 mm^2^, field of view: 488x716 mm using a 256x256 matrix, repetition time: 4.06 ms and echo time: 1.26x2.49 ms; flip angle 9° [19]. For all further analyses, water images were used.

### Deep learning framework for liver shape analysis

We developed a fully automatic framework to quantify radiomic shape features of the liver on T1-weighted MRI. The framework comprised the following steps: 1) deep learning model for 3D liver segmentation. The only input to the model was a T1-weighted isotropic VIBE-Dixon gradient-echo sequence using the water contrast reconstruction and the output was a 3D segmentation mask of the liver. 2) Quantification of radiomic shape features based on the 3D liver segmentation mask of the deep learning model. Radiomic features were extracted using PyRadimoics (https://pyradiomics.readthedocs.io/). The entire framework was implemented in NORA (www.nora-imaging.com), an open-source medical imaging platform.

### Deep learning model for liver segmentation

For automated liver segmentation, we employed a hierarchical, multiscale 3D convolutional neural network, which uses nested patches of fixed matrix size (https://bitbucket.org/reisert/patchwork/) [20]. In each scale, a UNet-like architecture is used, where the matrix size of the UNet is always of size 32^3^ voxels for all scales. We used a scale pyramid of depth four, where the patch size of the coarsest scale is a cube with side length of 80% of the full image. The physical size of the finest patch is selected such that a resolution 1.5, 1.5, 3 mm is achieved at the final output. As input, we stacked the derived water images of the T1-weighted Dixon sequence. The architecture of the basis UNet is close to the method described by Çiçek et al., 2016 [21], with feature dimensions (8,16,16,32,64) and max-pooling in the encoding layers and transposed convolutions in the decoding layers. Each UNet has n+8 output channels, where the first n is the number of labels and are used for intermediate loss computations. The logits of the total n+8 outputs are just forwarded as input to the next scale. The network is trained with the Adam optimizer with a learning rate 0.001. All labels are trained with ordinary binary crossentropy per channel. The prediction of a full volume was performed on a 16-core machine without GPU support and takes less than 5 minutes. All data management and processing were performed using NORA (www.nora-imaging.com).

For model development, a random subset of 124 participants was used. Segmentation labels of the liver were manually generated by a trained radiologist and proofed read/adapted if necessary, by an additional board-certified radiologist. For model training, a random subset of 80 participants was used. Independent testing was performed in the remaining, held-out 44 participants not seen during any part of training. Model performance was evaluated using the DICE index, the average surface distance and Pearsons´s correlation coefficient.

Subsequently, the model was applied to all participants of the KORA-MRI study. For additional quality control, the 3D liver segmentation masks of all participants were visually validated by a trained radiologist and excluded from further analyses, if substantial errors were detected.

### Radiomic feature extraction

Based on the 3D liver segmentation mask we extracted 14 radiomic shape features (e.g. volume, sphericity) using the open-source software PyRadiomics (version 3.0). Detailed descriptions of the individual features are provided on the PyRadiomics homepage (https://pyradiomics.readthedocs.io/en/latest/features.html).

### Assessment of impaired glucose metabolism and other clinical covariates

Baseline demographics and cardiometabolic risk factors of the study participants were evaluated in detailed standardized interviews and standardized medical examination as described previously in detail [18]. Variables of interest included age in years, sex, body mass index (BMI; kg/m^2^), weight (kg), height (cm), waist circumference (cm), hypertension (mmHg, defined as systolic blood pressure of at least 140 mmHg, diastolic blood pressure of at least 90 mmHg), antihypertensive medication, glycated haemoglobin (HbA1c %), total cholesterol (mg/dl), low density lipoprotein-C (mg/dl), triglycerides, (mg/dl), hepatic steatosis (defined as MRI-derived hepatic fat fraction >5% [19]), alcohol consumption (g/day) and smoking status (never, former, current).

### Outcome

The primary outcome of this study was impaired glucose metabolism defined according to the 1998 World Health Organization criteria [22] and assessed via an oral glucose tolerance test. Per study protocol, individuals with a 2-h serum glucose concentration >200 mg/dl and/or a fasting glucose level >125 mg/dl were classified as having type 2 diabetes, participants with a 2-h serum glucose concentration between 140 and 200 mg/dL; and/or an impaired fasting glucose concentration between 110 and 125 mg/dL as having prediabetes [19]. Participants with previously established and physician-validated type 2 diabetes did not undergo oral glucose test but were immediately classified as having type 2 diabetes.

### Statistical analysis

All statistical analysis was conducted using R version 4.1.2 and Microsoft Excel version 16.66.1. Continuous variables are described as mean±SD (standard deviation) or median and interquartile range (IQR) as appropriate. Categorical variables are provided as frequencies and percentages, respectively. Differences between demographics and cardiometabolic risk factors were tested using Student´s t-test, Wilcoxon rank-sum text or x2-test as appropriate.

To investigate the association between radiomic shape features of the liver and impaired glucose metabolism, a two-step approach was conducted. 1) Clustering approach: to test the combined association of all 14 extracted shape features of the liver and impaired glucose metabolism, we used k-medoids clustering (3 clusters) with a dissimilarity matrix based on Euclidean distance to derive unbiased data-driven clusters from the shape features only. Clusters were used as categorical exposures (reference: first cluster) in an ordinal logistic regression model with proportional odds assumption and outcomes prediabetes and diabetes. We estimated univariable and multiple adjusted Odds Ratios (OR), with stepwise adjustment for baseline demographics (age, sex, BMI) and cardiometabolic risk factors (total cholesterol, alcohol consumption, hypertension, smoking status, hepatic steatosis). 2) Individual approach: to investigate the association between individual shape features and impaired glucose metabolism, they were used as continuous exposures in ordinal logistic regression with adjustments as outlined above. Odds Ratios are reported per 1 SD change in shape feature. Liver volume was defined as the key feature of interest, as it is often estimated in clinical routine and easier to grasp than e.g. elongation or sphericity. To limit the analyses to features with the highest effect size and avoid redundancy with liver volume, only features with a univariable OR ≥1.5 and a correlation ≤0.5 with liver volume were considered. All p-values are two-sided and are considered to denote statistical significance if <0.05.

## Results

### Study population

**Fig 1:**
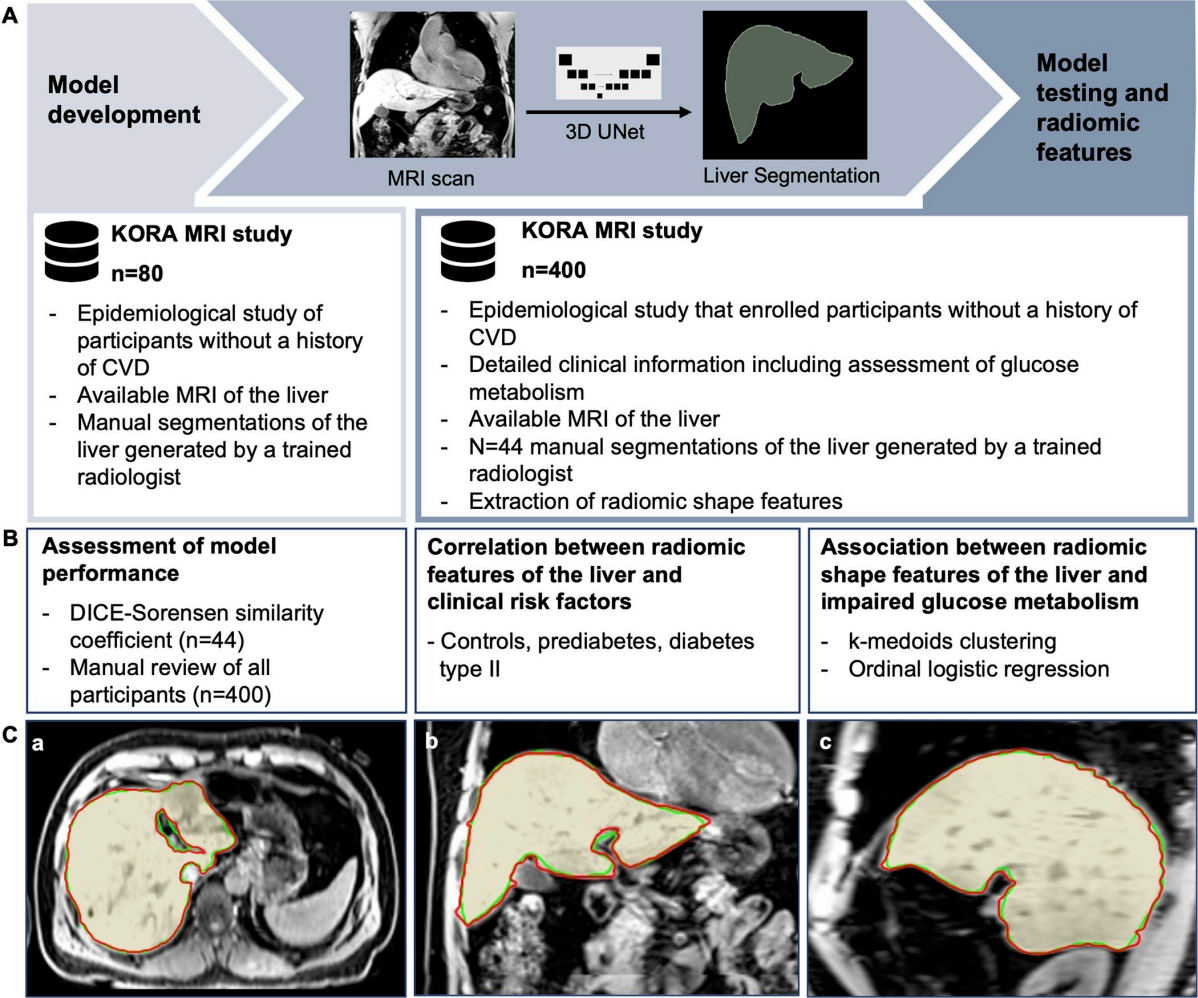
Overview of the study design. A) The deep learning framework for fully automated extraction of liver shape features was developed in participant of the KORA MRI study. B) The performance of the framework was tested in an independent random subset of 44 participants not seen during any part of model development. In addition, the predictive value of liver shape features for impaired glucose metabolism was investigated in the entire study cohort. C) Representative case of the independent testing dataset with manual (red) and automatically (green) generated liver segmentations. KORA = Cooperative Health Research in the Region of Augsburg; CVD = Cardiovascular Disease; MRI = Magnetic Resonance Imaging.

**Fig 2:**
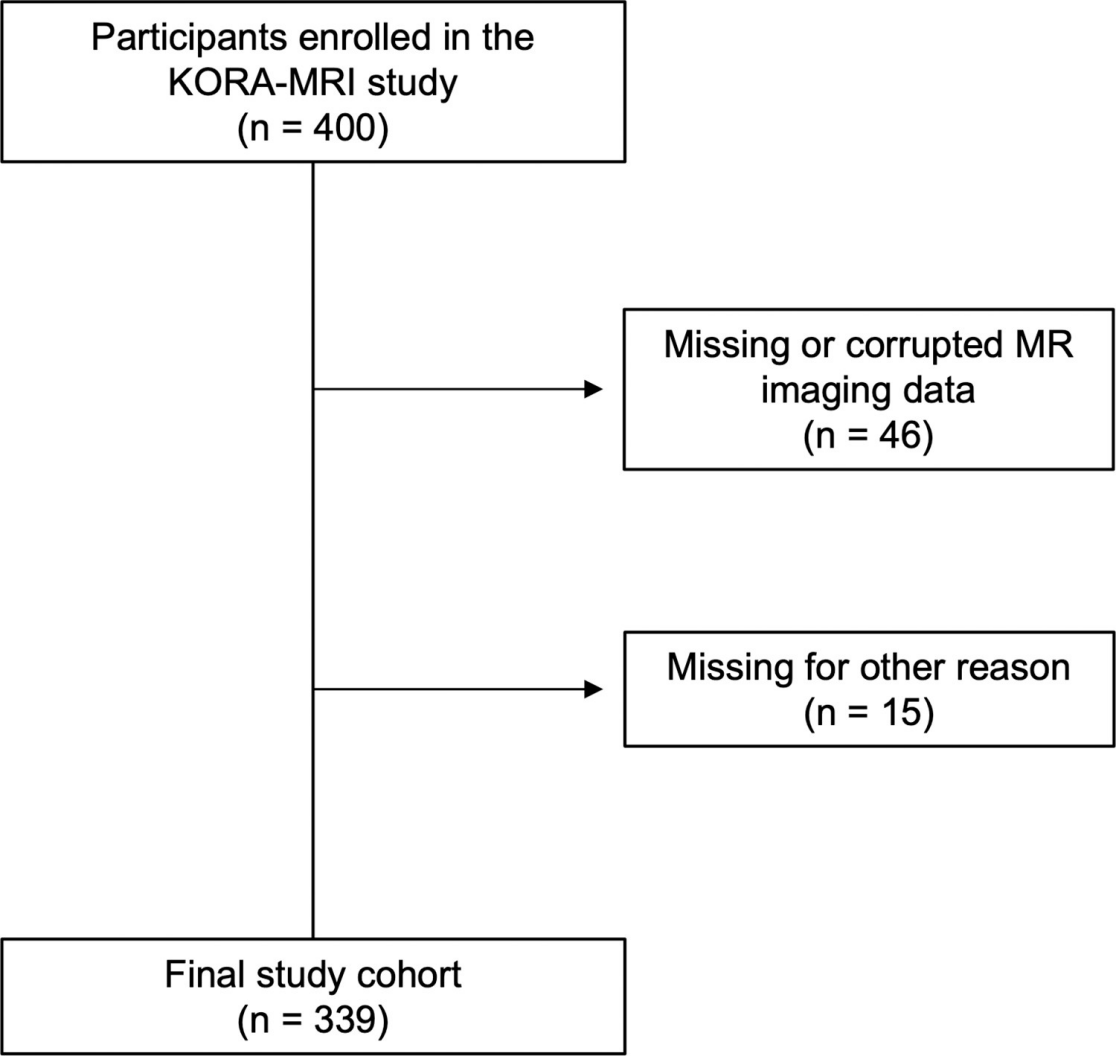
Participant flowchart of the KORA-MRI study. Other reason: 1 withdrew consent; 4 missing values of liver shape; 4 missing values of hepatic steatosis; 6 missing covariates.

An overview of the study design is presented in Fig 1. Of the 400 participants included in the KORA-MRI study, 46 participants were excluded due to missing or corrupted imaging data. Additional 15 participants were excluded due to missing covariates resulting in a final study cohort of 339 individuals (Fig 2) with a mean age of 56.3±9.1 years. 58.1% were male and 23.3% (79/339) vs. 13.6% (46/339) were classified as having prediabetes and type 2 diabetes, respectively. Participants with type 2 diabetes were significantly older, more likely men and presented more often with prevalent hypertension and hepatic steatosis compared to normoglycemic controls (all p<0.001). Further detailed demographics are provided in Table 1.

**Table 1:** Baseline demographics and cardiovascular risk factors.

Variable	Entire cohort	Normoglycemia	Prediabetes	Diabetes	p-value
	N = 339	N = 214 (63.1%)	N = 79 (23.3%)	N = 46 (13.6%)	
Mean age, years±SD	56.3 ± 9.1	54.1 ± 8.8	58.6 ± 8.6	62.5 ± 7.6	<0.001
Male sex	197 (58.1%)	108 (50.5%)	53 (67.1%)	36 (78.3%)	<0.001
BMI, kg/m^2^	28.0 ± 4.7	26.7 ± 4.3	30.4 ± 4.4	29.6 ± 4.9	<0.001
Weight, kg	82.9 ± 16.3	78.8 ± 15.8	91.1 ± 13.9	87.5 ± 16.5	<0.001
Height, cm	171.9 ± 9.7	171.4 ± 10.4	173.2 ± 8.8	171.7 ± 7.8	0.391
Waist circumference, cm	98.3 ± 13.8	93.8 ± 12.8	106.0 ± 10.8	106.2 ± 13.5	<0.001
Hypertension	112 (33.0%)	45 (21.0%)	36 (45.6%)	31 (67.4%)	<0.001
Antihypertensive medication	82 (24.2%)	36 (16.8%)	25 (31.6%)	21 (45.7%)	<0.001
HbA1c, %	5.6 ± 0.8	5.3 ± 0.3	5.6 ± 0.3	6.8 ± 1.4	<0.001
Total Cholesterol, mg/dL	218.8 ± 37.3	216.4 ± 36.1	227.2 ± 32.1	215.2 ± 48.0	0.068
LDL-C, mg/dL	140.6 ± 33.9	138.9 ± 32.0	148.0 ± 31.8	135.6 ± 43.3	0.072
Triglycerides, mg/dL	131.0 ± 85.7	105.3 ± 60.7	158.7 ± 85.6	202.8 ± 123.5	<0.001
Hepatic Steatosis	188 (55.5%)	85 (39.7%)	64 (81.0%)	39 (84.8%)	<0.001
Alcohol consumption, g/day [median (CI)]	8.6 [0.5, 26.8]	6.6 [0.4, 25.4]	17.1 [5.7, 40.9]	5.3 [0.0, 25.6]	0.013
Never smoker	122 (36.0%)	83 (38.8%)	24 (30.4%)	15 (32.6%)	0.058
Ex-smoker	149 (44.0%)	83 (38.8%)	39 (49.4%)	27 (58.7%)	
Smoker	68 (20.1%)	48 (22.4%)	16 (20.3%)	4 (8.7%)	

Values are given as arithmetic mean ± standard deviation for continuous variables, unless indicated otherwise. Categorical data are given as counts (percentage). P-values from one-way ANOVA, Kruskal-Wallis Test or χ2-Test, where appropriate. BMI = Body Mass Index; HbA1c = Glycated Haemoglobin; LDL = Low Density Lipoprotein

### Deep learning framework for liver shape analysis

**Fig 3:**
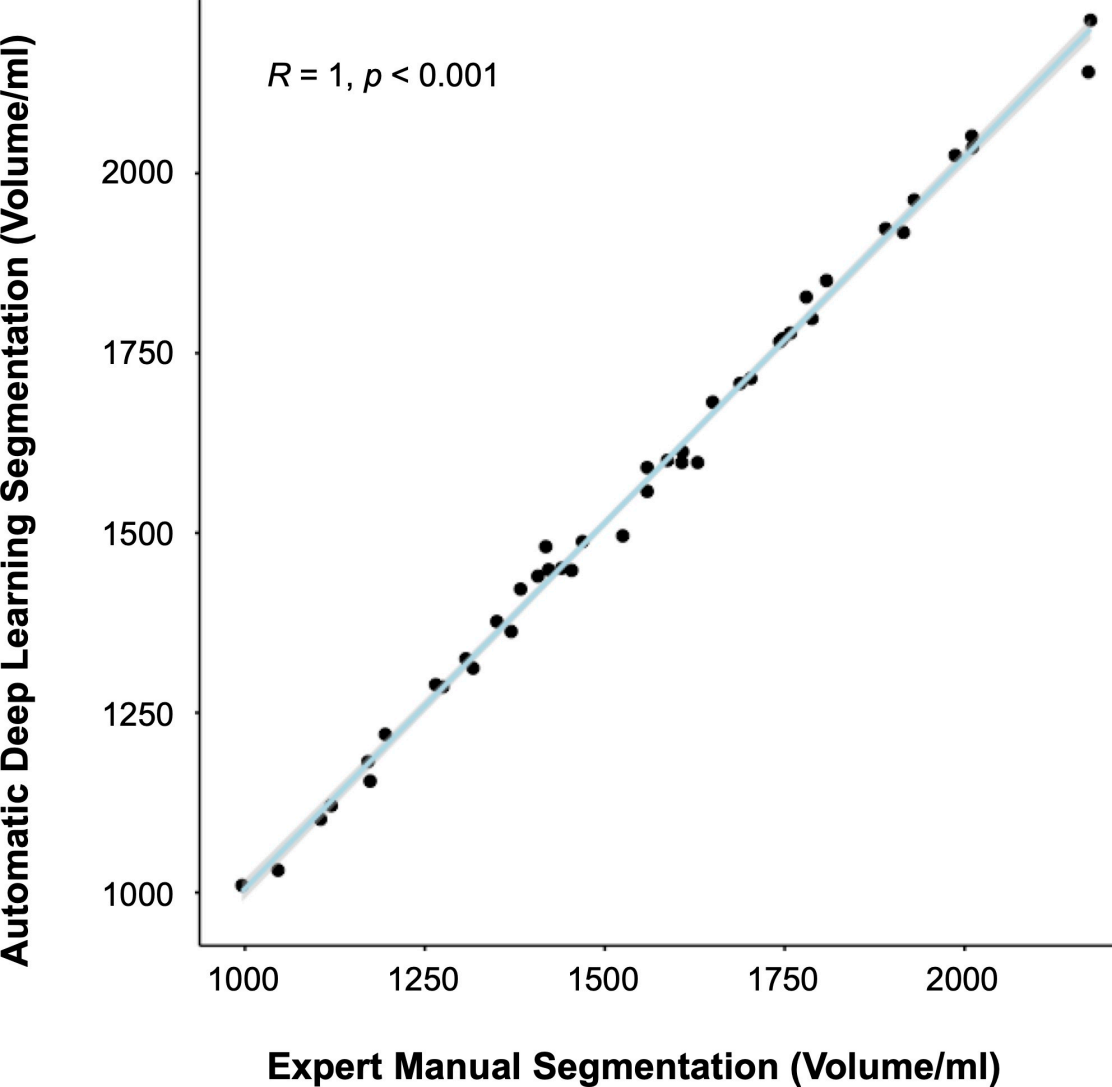
Pearson’s correlation between expert manual vs. automatic deep learning segmentations. Pearson’s correlation between expert manual and automatic deep learning segmentations of the liver indicating a high model performance with a correlation coefficient of r = 1.0 (p<0.001) in the independent testing dataset.

In the independent testing dataset, the deep learning liver segmentation model showed a high performance with a mean Dice score of 97.0±0.01, an average surface distance of a 0.82±0.19 mm and a Pearson´s correlation coefficient of 0.998 (p<0.001) (Fig 3) compared to the manual labels generated by the expert radiologist.

Visual inspection of all participants revealed no systemic segmentation errors or major failures. From all segmentation masks, the entire panel of 14 radiomic shape features were successfully extracted. An overview and a correlation matrix of the features is provided in S1 Fig.

### Association between liver shape features and impaired glucose metabolism

Clustering approach: First, we investigated the association between all 14 shape features and impaired glucose metabolism using a cluster-based approach. The 3 identified clusters comprised n = 115 (cluster 1), n = 138 (cluster 2) and n = 86 (cluster 3) participants, respectively. Participants in the high-risk cluster (cluster 3) were more likely men, presented with hypertension and hepatic steatosis compared to cluster 1 and 2, (all p<0.001).

**Fig 4:**
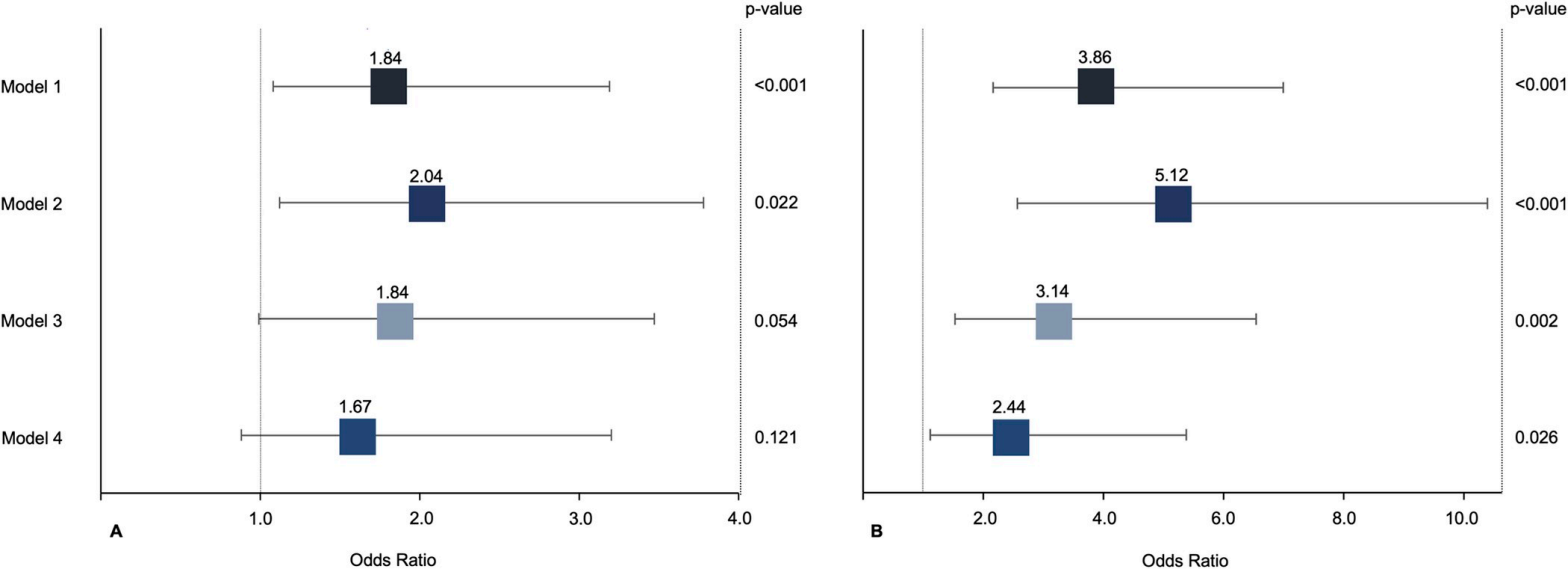
Association between hepatic shape feature clusters and impaired glucose metabolism. Association between hepatic shape features clusters (A = intermediate risk cluster; B = high-risk cluster) and impaired glucose metabolism (prediabetes or diabetes) from ordinal logistic regression with proportional odds assumption. Model 1 = univariate; Model 2 = adjusted for age, sex; Model 3 = adjusted age, sex and hepatic steatosis; Model 4 = adjusted for age, sex, BMI, alcohol consumption, hypertension, smoking (never, ex, current), total cholesterol and hepatic steatosis. Boxes indicate odds ratios; lines 95% confidence intervals. BMI = Body Mass Index.

On a univariable level, there was an independent association between cluster 2 and cluster 3 and impaired glucose metabolism (OR 1.84 [95% CI 1.08–3.19] and OR 3.86 [95% CI 2.17–7.00]; p < 0.001) compared to cluster 1. The association remained robust after adjustment for age and sex Fig 4. After further adjustment for the complete panels of cardiometabolic risk factors, only the OR for cluster 3 remained significant (OR 2.44 [95% CI 1.12–5.38]; p = 0.026) whereas the signal for cluster 2 was attenuated (OR 1.67 [95% CI 0.88–3.20]; p = 0.121). A summary is provided in Fig 4.

**Fig 5:**
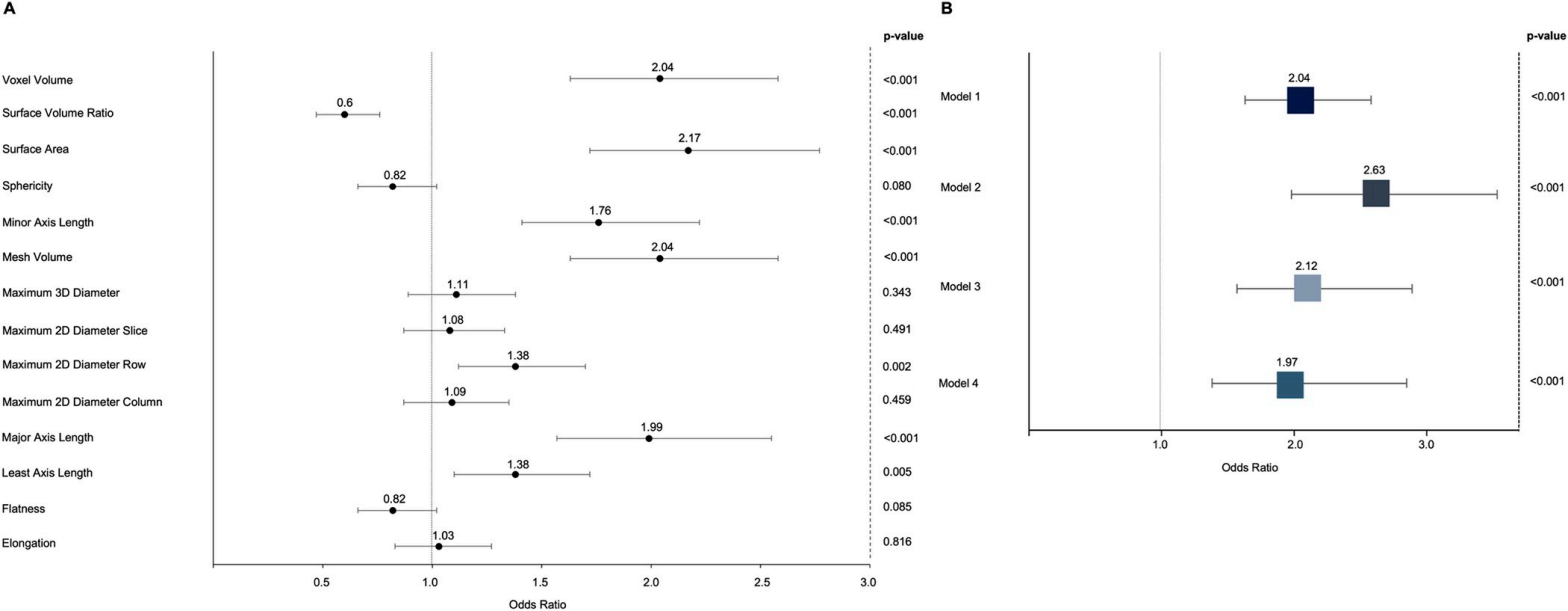
Association between individual hepatic shape features with impaired glucose metabolism and association between liver volume and impaired glucose metabolism. A) Association between individual hepatic shape features and impaired glucose metabolism (prediabetes or diabetes) from ordinal logistic regression with proportional odds assumption. Figure depicts univariate odds ratios with 95% confidence intervals for hepatic shape features association with impaired glucose metabolism. B) Association between liver volume and impaired glucose metabolism (prediabetes or diabetes) from ordinal logistic regression with proportional odds assumption. Model 1 = univariate; Model 2 = adjusted for age, sex; Model 3 = adjusted age, sex and hepatic steatosis; Model 4 = adjusted for age, sex, BMI, alcohol consumption, hypertension, smoking (never, ex, current), total cholesterol and hepatic steatosis. Boxes indicate odds ratios; lines 95% confidence intervals. BMI = Body Mass Index.

Individual approach: Next, we investigated the association between individual shape features and impaired glucose metabolism. A summary and correlation matrix of the 14 extracted features are given in S1 Fig and S1 Table. Surface Area, Voxel Volume, Least-, Minor-, Major Axis Length, Surface Volume Ratio, Maximum 2D Diameter Row and Mesh Volume were significantly associated with impaired glucose metabolism while no significant association was found for Elongation, Sphericity, Flatness, Maximum2D Diameter Column, Maximum 2D Diameter Slice and Maximum 3D Diameter (Fig 5). Applying the feature reduction approach described above (OR ≥1.5 and correlation with liver volume ≤0.5), liver volume remained as the only feature independently associated with impaired glucose metabolism on a univariable level (OR 2.04 [95% CI 1.63–2.50]; p = 0.001). This association remained significant after adjustment for age and sex and the complete panel of cardiometabolic risk factors (OR 1.97 [1.38–2.85]; p<0.001) (Fig 5).

## Discussion

In this study, we developed a fully automatic deep learning framework to investigate the association between radiomic shape features of the liver and impaired glucose metabolism. Our results demonstrate that MRI-derived radiomic shape features of the liver are independently associated with impaired glucose metabolism after adjustment for traditional cardiometabolic risk factors and hepatic steatosis in a cohort of individuals with prediabetes, type 2 diabetes and normal controls.

Currently, several accurate and cheap tests exist for the diagnosis and monitoring of impaired glucose metabolism. Nevertheless, the number of individuals with undiagnosed diabetes is high with an estimate of 44.7% in 2021 [9]. This number is of great socioeconomic concern given the costs associated with the treatment of diabetes and its complications which are estimated by Bommer et al. to accumulate to 2.1 trillion dollars in 2030 [5]. Hence, screening and early detection are of great importance to identify individuals in an asymptomatic stage to reduce the need for intensified treatment and management of potentially irreversible complications since dietary changes and physical activity have proven to be sufficient preventive measures in the early disease stage [23]. Therefore, novel approaches to identify individuals at risk of type 2 diabetes are desirable to reduce morbidity and mortality.

In this context, opportunistic risk assessment based on routinely acquired but currently unused imaging data may provide a valuable solution. While vast amounts of imaging data are acquired every day, only a small fraction is used to rule out or confirm a suspected clinical finding. Quantification of prognostic imaging information, such as coronary artery calcium or liver fat is only done for specific indications requiring dedicated equipment and expertise [24] [25]. With recent advances in deep learning, automatic quantification of such information has become feasible at high speed and low additional cost. For example, Zeleznik et al. proposed a deep learning model for automatic quantification of coronary artery calcium and demonstrated a robust performance in more than 20.000 individuals [26]. Graffy et al. presented a method for population-based assessment of hepatic steatosis on CT imaging in more than 9000 subjects [27]. In line with these studies, our results demonstrated the potential for opportunistic screening of impaired glucose metabolism using a standard MRI of the liver in a cohort of 339 individuals. As MRI of the liver is frequently acquired in clinical routine, our framework could add an additional layer of information to the standard radiology report with a personalized risk estimate for impaired glucose metabolism. By implementing the model in the Picture Archiving and Communication System, this could be done with minimal disruption of current workflows and provide valuable prognostic information to the patient and treating physician that currently goes unnoticed. If an increased risk is detected, the patient could be notified to initiate risk factor assessment and lifestyle interventions.

The literature on opportunistic risk assessment of impaired glucose metabolism using medical imaging data is limited. We identified only two studies investigating the value of CT-based radiomic features of the liver and the pancreas to estimate the risk of diabetes [28,29]. In contrast to the current study, the identified features/signatures comprised various features, which are not intuitively understood and generalizability as well as stability to other populations is questionable. A more simple and known measure like liver volume identified in our analysis is easier to grasp for the referring physician and the patient alike, which might increase acceptance, as the black box characteristic of many deep learning approaches is a well-known drawback and limitation for clinical implementation [30,31].

Several limitations of this study need to be considered. First, our study population comprised predominantly Caucasian subjects without known cardiovascular disease. Whether our results generalize to other races/ethnicities and individuals with prevalent cardiovascular disease needs to be tested in more diverse populations. Further, the deep learning framework was not externally validated. Thorough testing and potential retraining/transfer learning might be necessary to ensure reliable performance in other populations. Moreover, we only explored the association between radiomic shape features of the liver. Whether higher-level features carry further additive value was beyond the scope of this study. Finally, although our approach identified individuals at risk of impaired glucose metabolisms it remains unknown whether this would change clinical workflows for disease prevention and patient management.

In conclusion, the proposed deep learning framework allows for identifying individuals at increased risk of impaired glucose metabolism on routinely acquired MRI of the liver independent of traditional cardiometabolic risk factors and hepatic steatosis. This approach could be used for opportunistic screening in daily care to prompt risk factor assessment and initiate preventive measures to reduce morbidity and mortality.

## Supporting information

S1 Table Radiomic shape parameters of our study population. (DOCX)

S2 Table Baseline demographics and cardiovascular risk factors for the clusters. (DOCX)

S1 Fig Pearson´s correlation coefficient between the extracted radiomic shape features of the liver. 1 indicates a perfect correlation. (TIF)
