# Supplementary material for: Deep learning to estimate impaired glucose metabolism from Magnetic Resonance Imaging of the liver: An opportunistic population screening approach
Source: PLOS Digit Health. 2024 Jan 16;3(1):e0000429. doi: 10.1371/journal.pdig.0000429 (PMC10791001; doi:10.1371/journal.pdig.0000429)
Supplement: S1 Table — (DOCX) [file pdig.0000429.s001.docx]

| Variable | Entire cohort | Normoglycemia | Prediabetes | Diabetes | p-value |
| --- | --- | --- | --- | --- | --- |
| Surface Area, mm² | 99151.1 ± 15285.3 | 95217.1 ± 13349.3 | 103676.0 ± 14030.6 | 109681.6 ± 18635.4 | <0.001 |
| Voxel Volume, mm³ | 1575769.2 ± 352126.1 | 1488309.8 ± 307802.2 | 1681443.7 ± 342910.8 | 1801160.8 ± 413699.6 | <0.001 |
| Elongation | 0.7 ± 0.1 | 0.7 ± 0.1 | 0.7 ± 0.1 | 0.7 ± 0.1 | 0.544 |
| Flatness | 0.5 ± 0.1 | 0.5 ± 0.1 | 0.5 ± 0.1 | 0.5 ± 0.1 | 0.217 |
| Least Axis Length, mm | 102.6 ± 10.4 | 101.4 ± 10.3 | 104.3 ± 9.8 | 105.2 ± 11.2 | 0.018 |
| Major Axis Length, mm | 194.6 ± 17.0 | 190.7 ± 15.7 | 199.2 ± 16.3 | 204.7 ± 18.3 | <0.001 |
| Maximum 2D Diameter Column, mm² | 233.0 ± 45.2 | 232.3 ± 47.2 | 229.4 ± 35.7 | 242.7 ± 49.9 | 0.266 |
| Maximum 2D Diameter Row, mm² | 216.1 ± 71.7 | 206.7 ± 68.3 | 232.4 ± 73.8 | 232.0 ± 76.8 | 0.006 |
| Maximum 2D Diameter Slice, mm² | 283.7 ± 86.2 | 280.8 ± 85.3 | 291.2 ± 86.1 | 284.2 ± 91.1 | 0.660 |
| Maximum 3D Diameter, mm³ | 319.3 ± 99.2 | 315.3 ± 96.0 | 327.1 ± 96.4 | 324.8 ± 118.3 | 0.613 |
| Mesh Volume, mm³ | 1575118.6 ± 352084.7 | 1487674.0 ± 307782.2 | 1680781.5 ± 342840.0 | 1800461.9 ± 413654.6 | <0.001 |
| Minor Axis Length, mm | 139.2 ± 15.1 | 136.5 ± 13.9 | 141.3 ± 14.0 | 148.5 ± 18.1 | <0.001 |
| Sphericity | 0.7 ± 0.0 | 0.7 ± 0.0 | 0.7 ± 0.0 | 0.7 ± 0.0 | 0.142 |
| Surface Volume Ratio | 0.1 ± 0.0 | 0.1 ± 0.0 | 0.1 ± 0.0 | 0.1 ± 0.0 | <0.001 |

S1 Table - Radiomic shape parameters of our study population

Values are given as arithmetic mean ­­± standard deviation. P-value from one-way ANOVA.
