# Supplementary material for: Deep learning to estimate impaired glucose metabolism from Magnetic Resonance Imaging of the liver: An opportunistic population screening approach
Source: PLOS Digit Health. 2024 Jan 16;3(1):e0000429. doi: 10.1371/journal.pdig.0000429 (PMC10791001; doi:10.1371/journal.pdig.0000429)
Supplement: S2 Table — (DOCX) [file pdig.0000429.s002.docx]

S2 Table - Baseline demographics and cardiovascular risk factors for the clusters

| Variable | Cluster I | Cluster II | Cluster III | p-value |
| --- | --- | --- | --- | --- |
| Participants N=339 | N= 115 (33.9%) | N= 138 (40.7%) | N= 86 (25.4%) |  |
| Mean age, years±SD | 57.6 ± 9.4 | 56.0 ± 9.5 | 54.8 ± 8.1 | 0.089 |
| Male sex | 35 (30.4%) | 89 (64.5%) | 73 (84.9%) | <0.001 |
| BMI, kg/m² | 26.7 ± 4.8 | 27.6 ± 3.7 | 30.8 ± 4.9 | <0.001 |
| Hypertension | 28 (24.3%) | 45 (32.6%) | 39 (45.3%) | 0.007 |
| Normoglycemia | 87 (75.7%) | 87 (63.0%) | 40 (46.5) | <0.001 |
| Prediabetes | 21 (18.3) | 34 (24.6%) | 24 (27.9%) |  |
| Diabetes | 7 (6.1%) | 17 (12.3%) | 22 (25.6%) |  |
| HbA1c, % | 5.5 ± 0.5 | 5.5 ± 0.9 | 5.7 ± 0.8 | 0.079 |
| Total Cholesterol, mg/dL | 226.5 ± 37.2 | 215.1 ± 34.3 | 214.4 ± 40.6 | 0.023 |
| LDL-C, mg/dL | 144.3 ± 35.8 | 138.7 ± 30.1 | 138.7 ± 36.7 | 0.0349 |
| Triglycerides, mg/dL | 104.1 ± 52.0 | 122.5 ± 74.5 | 180.4 ± 114.3 | <0.001 |
| Hepatic Steatosis | 43 (37.4%) | 69 (50.0%) | 76 (88.4%) | <0.001 |
| Alcohol consumption, g/day | 13.5 ±18.0 | 17.7 ± 21.0 | 28.0 ± 32.8 | <0.001 |
| Never smoker | 50 (43.5%) | 51 (37.0%) | 21 (24.4%) | 0.085 |
| Ex-smoker | 43 (37.4%) | 60 (43.5%) | 46 (53.5%) |  |
| Smoker | 22 (19.1%) | 27 (19.6%) | 19 (22.1%) |  |

Values are given as arithmetic mean ± standard deviation for continuous variables, unless indicated otherwise. Categorical data are given as counts (percentage). P-values from one-way ANOVA, Kruskal-Wallis Test or χ2-Test, where appropriate. BMI=Body Mass Index; HbA1c=Glycated Haemoglobin; LDL=Low Density Lipoprotein
