# Supplementary figures and images for: Deep learning to estimate impaired glucose metabolism from Magnetic Resonance Imaging of the liver: An opportunistic population screening approach
Source: PLOS Digit Health. 2024 Jan 16;3(1):e0000429. doi: 10.1371/journal.pdig.0000429 (PMC10791001; doi:10.1371/journal.pdig.0000429)

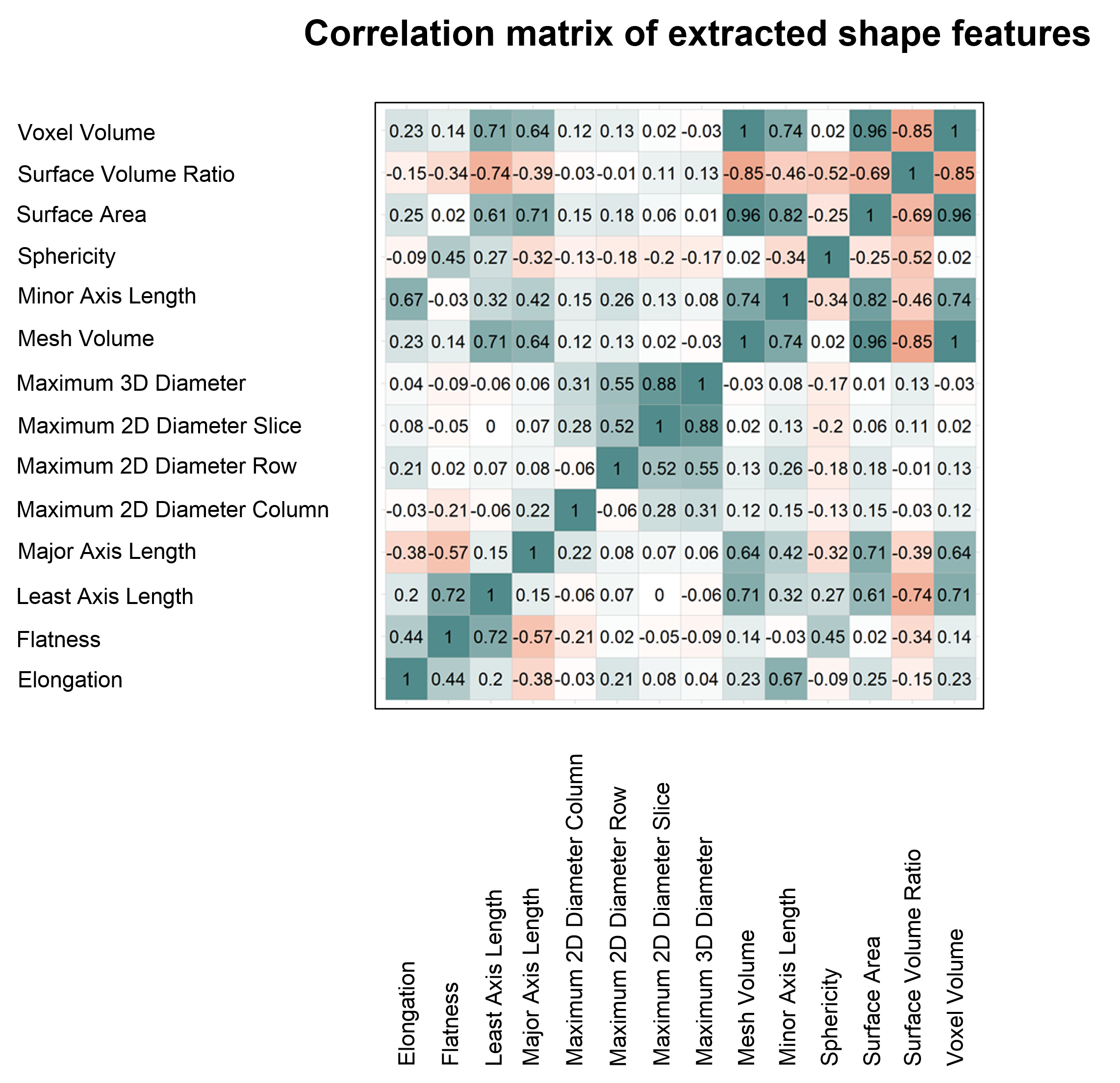

Supplement: S1 Fig — (TIF) [file pdig.0000429.s003.tif]
